# Weight gain from early to middle adulthood increases the risk of incident asthma later in life in the United States: a retrospective cohort study

**DOI:** 10.1186/s12931-021-01735-7

**Published:** 2021-05-05

**Authors:** Tao Wang, Yunping Zhou, Nan Kong, Jianzhong Zhang, Guo Cheng, Yuxin Zheng

**Affiliations:** 1 School of Public Health, Qingdao University, 308 Ningxia Road, Qingdao, 266071 Shandong People’s Republic of China; 2 School of Nursing, Qingdao University, 308 Ningxia Road, Qingdao, 266071 Shandong People’s Republic of China; 3 Laboratory of Molecular Translational Medicine, Centre for Translational Medicine, Key Laboratory of Birth Defects and Related Diseases of Women and Children (Sichuan University), Ministry of Education, West China Second University Hospital, Sichuan University, Chengdu, Sichuan People’s Republic of China

**Keywords:** Asthma, Body mass index, Obesity, Weight gain

## Abstract

**Background:**

Data describing the effects of weight change across adulthood on asthma are important for the prevention of asthma. This study aimed to investigate the association between weight change from early to middle adulthood and risk of incident asthma.

**Methods:**

Using data from the National Health and Nutrition Examination Survey (NHANES), we performed a nationally retrospective cohort study of the U.S. general population. A total of 20,771 people aged 40–74 years with recalled weight at young and middle adulthood were included in the cohort. Four weight change groups were categorized: stable non-obesity, non-obesity to obesity, obesity to non-obesity, and stable obesity. Hazard ratios (HRs) and 95% confidence intervals (CIs) relating weight change to incident asthma over 10 years of follow-up were calculated using Cox models adjusting for covariates.

**Results:**

Compared with the stable non-obesity group, the HRs of incident asthma were 1.63 (95% CI = 1.29 to 2.07, *P* < 0.001) for the non-obesity to obesity group, 1.41 (95% CI = 0.97 to 2.05, *P* = 0.075) for stable obesity group, and 1.21 (95% CI = 0.41 to 3.62, *P* = 0.730) for the obesity to non-obesity group. In addition, participants who gained more than 20 kg from young to middle adulthood had a HR of 1.53 (95% CI = 1.15 to 2.03, *P* = 0.004), compared with those whose weight remained stable (weight change within 2.5 kg).

**Conclusions:**

Weight gain from early to middle adulthood was associated with higher risk of incident asthma as compared to those who maintained normal weight. Thus, maintaining normal weight throughout adulthood might be important for the primary prevention of adult-onset asthma.

**Supplementary Information:**

The online version contains supplementary material available at 10.1186/s12931-021-01735-7.

## Introduction

Asthma and obesity are major public health problems that have increased in the past decades. From 1999 through 2018, the age-adjusted prevalence of obesity among adults increased from 30.5 to 42.4% in the United States [1]. Although asthma was less prevalent than obesity, it also increased from 7.1 to 9.2% between 2001 and 2014 [2]. A meta-analysis of seven prospective studies involving more than 300 000 adults found a dose–response relationship between obesity and asthma, the odds ratio of incident asthma was 1.38 in the overweight and 1.92 in the obesity groups compared with normal weight [3]. Results from Mendelian Randomization studies provided causal evidence for obesity increasing the risk of asthma [4–6].

However, many previous cohort studies [7–10] included only a single measurement of Body Mass Index (BMI), which ignored the dynamic feature of body weight over time. Thus, more studies are needed to assess the long-term consequences of weight change during certain life periods. A meta-analysis including 147,252 European children in 31 birth cohort studies found that rapid weight gain in infancy was positively associated with childhood asthma [11]. The Taiwan Children Health Study conducted by Chen et al. indicated that rapid adiposity growth might increase the incidence or recurrence of symptoms of childhood asthma [12]. In contrast to childhood-onset asthma, less is known about the factors associated with adult-onset asthma, particularly from longitudinal studies [13]. Asthma that began in adulthood was often non-atopic, more severe and associated with a faster decline in lung function compared with childhood-onset asthma [13].

Excess adiposity tends to accrue during early and middle adulthood for most people. Adult weight gain has been associated with increased mortality risk, diabetes, hypertension, cardiovascular disease, several types of cancer, etc. [14–17] However, studies exploring the effect of weight change from early to middle adulthood on adult-onset asthma were limited. The National Health and Nutrition Examination Survey (NHANES) has routinely collected information about participants’ history of weight (weight at age 25 and weight at 10 years before the survey) and asthma diagnosis. Leveraging these data provided an opportunity to investigate the relationship between weight changes, in the form of weight at age 25 and weight 10 years before the survey, and the incidence of asthma during 10 years of follow-up before the NHANES survey. Therefore, this study aimed to examine the relations of weight change from early (age 25 years) to middle adulthood (mean age 44 years) with asthma incidence in the United States. We conducted this work from an evidence-based medicine perspective.

## Methods

### Study design and population

Details of National Health and Nutrition Examination Survey (NHANES) have been described elsewhere [18]. NHANES is a series of ongoing cross-sectional surveys conducted by the National Center for Health Statistics (NCHS) of the Centers for Disease Control and Prevention (CDC). Representative samples of the non-institutional U.S. population were selected by a complex stratified, multistage probability sampling design. NHANES was approved by the National Center for Health Statistics research ethics review board, and written informed consent from all the participants was provided during the survey. Because the data are publicly available and de-identified, institutional review board approval was not required for this analysis.

This study used data across 9 cycles of the continuous NHANES (1999–2000 through 2015–2016) including adults aged 40–74 [15] at examination. We used recall questions on weight history and age at asthma diagnosis to create a retrospective cohort from the cross-sectional data. Specifically, self-reported weight change was assessed by participant recall of weight at age 25 and 10 years before the NHANES survey. We defined baseline as 10 years before the survey. Incident asthma was determined from respondents affirming that a health care provider had indicated a diagnosis of asthma. The reported age (years) at diagnosis was used to establish the time of asthma onset. The design method for retrospective cohort study has been described in detail in a previous publication using NHANES data [14, 15, 19, 20]. The study design was visually depicted in Additional file 1: Figure S1.

Participants who reported a date of onset that was before the initiation of follow-up were considered prevalent asthma cases and were excluded. We also excluded participants missing information of asthma diagnosis, those without diagnosis time and those without BMI at age 25 years and (or) at age 10 years before survey. Finally, a sample size of 20,771 individuals remained in our cohort for analysis (Additional file 1: Figure S2).

### Assessments of weight change

Data on weight at age 25 years and at 10 years before the NHANES survey were recalled. Measured height at the examination was used to calculate BMI, unless the participant was 50 years or older at the time of the survey. In this case, reported height at 25 was used to calculate BMI at 25, and measured height at the examination was used to calculate BMI at 10 years before the survey [15]. We categorized each of the two BMI variables into three groups: underweight and normal weight (< 25.0), overweight (25.0–29.9), and obesity (≥ 30.0) [21].

BMI change categories were then generated to capture weight change over the life course of an individual. We defined four weight change patterns based on BMI (kg/m^2^) at age 25 and on BMI 10 years prior to the survey: stable non-obesity pattern (BMI_age 25_ < 30 kg/m^2^ and BMI_10 years prior_ < 30) (reference group), non-obesity to obesity pattern (BMI_age 25_ < 30 kg/m^2^ and BMI_10 years prior_ ≥ 30), obesity to non-obesity pattern (BMI_age 25_ ≥ 30 kg/m^2^ and BMI_10 years prior_ < 30), stable obesity pattern (BMI_age 25_ ≥ 30 kg/m^2^ and BMI_10 years prior_ ≥ 30). We also classified absolute weight change in each time interval into five groups: weight loss of at least 2.5 kg, weight change within 2.5 kg (reference group), weight gain of at least 2.5 kg but less than 10.0 kg, weight gain of at least 10 kg but less than 20.0 kg, and weight gain of at least 20.0 kg. These weight change categories were comparable with those used in previous studies [14–16, 20].

### Covariates

Information on covariates was available through questionnaires, including age, gender, race/ethnicity (Mexican American, Other Hispanic, Non-Hispanic White, Non-Hispanic Black, Other), education level (less than high school, high school or equivalent, and college or above), family income-poverty ratio (≤ 1.3, ~ 1.85, ~ 3.0, and > 3.0), smoking status (ever and never smokers), and family history of asthma.

### Statistical analysis

All analyses incorporated the sample weights, stratification, and clustering of the complex sampling design to ensure nationally representative estimates. Descriptive statistics were computed for all variables. For continuous variables, means and 95% confidence intervals (CIs) were calculated. For categorical and binary variables, percentages were depicted. We used Cox proportional hazards models with time in study as the underlying time metric to estimate the hazard ratios (HRs) and corresponding 95% CIs for incident asthma in relation to weight change patterns from age 25 years to 10 years before survey.

For the main analyses, we examined the associations between the four weight change patterns and developing asthma. The stable non-obesity pattern was used as the reference to which all other weight change patterns were compared. We adjusted for baseline age, gender, and race/ethnicity in model 1. We further adjusted for education level, family income-poverty ratio level, smoking status, and family history of asthma in model 2. Dummy variables were used to indicate missing data for the covariates. Predefined subgroup analyses and potential effect modifications were conducted by baseline age (< 50 and ≥ 50 years), gender (Male and Female), smoking status (Ever and Never smoking), and family history of asthma (Yes and No). No adjustment for multiplicity in the subgroup analysis were done because of the exploratory design of this study part.

A secondary analysis was implemented with five weight change patterns. Those originally in the stable non-obesity pattern were categorized into two new groups: stable normal pattern (BMI_age 25_ < 25.0 kg/m^2^ and BMI_10 years prior_ < 25.0), and maximum overweight pattern (25.0–29.9 kg/m^2^ at either time but not ≥ 30.0 at the other time). Stable normal pattern was used as the reference category for this model.

We also investigated the associations between absolute weight change groups and incident asthma risk, as well as the linear dose–response relation. For test of trend, we calculated the association with incident asthma by treating the categories of absolute weight change as ordinal variables. In addition, the absolute weight changes were also treated as continuous variables to investigate the robustness of our results.

Finally, we calculated population attributable fractions (PAFs) of incident asthma owing to weight change using the following formula [22]:

$$PAF = \frac{{\sum\limits_{i = 1}^{k} {p_{i} (HR_{i} - 1)} }}{{1 + \sum\limits_{i = 1}^{k} {p_{i} (HR_{i} - 1)} }}$$

where *p*_*i*_ is the proportion of weight change pattern *i*, *HR*_*i*_ is the hazard ratio of incident asthma in weight change pattern *i*, and *k* is the total number of weight change patterns. A given PAF represents the proportion of incident asthmas that could be reduced if people with a particular weight change pattern were redistributed to another pattern and experienced the same relative risks as individuals in that new pattern.

All statistical analyses were conducted in 2020 using SAS 9.4 (SAS Institute Inc., Cary, NC, USA) [23]. The forest plot was made by the “forestplot” package [24] in R 4.0.2 (R Foundation for Statistical Computing, Vienna, Austria) [25]. Statistical significance was defined as *P* < 0.05 using two-sided tests.

## Results

### Baseline characteristics and weight change pattern

Table 1 reported characteristics of the sample with weighted estimates and unweighted sample sizes stratified by weight change category. The mean age of the sample was 44.2 years at baseline, and 50.7% were female. The study sample was 6.0% Mexican American, 4.3% other Hispanic, 73.4% non-Hispanic white, 10.3% non-Hispanic black and 6.0% others. The mean BMI was 23.5 kg/m^2^ at age 25, and 27.1 kg/m^2^ at baseline. On average, participants gained 9.4 kg weight from age 25 years to baseline.

**Table 1:** Characteristics of study participants in NHANES 1999–2016 according to weight change patterns from age 25 years to 10 years before survey

Characteristics	Stable non-obesity	Non-obesity to obesity	Obesity to non-obesity	Stable obesity	Total
Participants	15,293 (75.2)	4015 (18.0)	209 (0.9)	1254 (5.9)	20,771
Mean (95% CI) baseline age, years^a^	43.9 (43.6, 44.1)	46.9 (46.4, 47.3)	41.0 (39.7, 42.3)	41.4 (40.7, 42.1)	44.2 (44.0, 44.5)
Female	7621 (52.0)	1965 (47.7)	83 (40.8)	598 (44.4)	10,267 (50.7)
Race/ethnicity					
Mexican American	2542 (5.6)	824 (7.0)	54 (8.4)	229 (7.4)	3649 (6.0)
Other Hispanic	1243 (4.5)	314 (3.6)	20 (7.8)	86 (3.6)	1663 (4.3)
Non-Hispanic White	6854 (73.4)	1773 (74.4)	87 (70.5)	510 (70.5)	9224 (73.4)
Non-Hispanic Black	3177 (9.7)	970 (11.4)	43 (11.8)	393 (15.6)	4583 (10.3)
Other	1477 (6.9)	134 (3.6)	5 (1.5)	36 (2.8)	1652 (6.0)
Education^b^					
Less than high school	4029 (16.0)	1160 (17.3)	82 (22.5)	348 (16.0)	5619 (16.3)
High school or equivalent	3526 (23.4)	983 (26.0)	41 (19.6)	320 (27.0)	4870 (24.0)
College or above	7730 (60.6)	1870 (56.7)	86 (57.8)	585 (57.0)	10,271 (59.7)
Family income-poverty ratio level^b^					
0 ~ 1.3	3521 (15.0)	1017 (17.0)	75 (28.3)	359 (19.9)	4972 (15.8)
~ 1.85	1618 (8.0)	453 (9.4)	26 (10.2)	146 (10.7)	2243 (8.4)
~ 3	2448 (16.2)	727 (19.0)	32 (14.9)	219 (17.6)	3426 (16.8)
> 3	6362 (60.8)	1496 (54.6)	67 (46.6)	430 (51.7)	8355 (59.0)
Ever smoker^a^	7286 (48.6)	1880 (48.4)	133 (62.5)	545 (43.3)	9844 (48.4)
Family history of asthma	1668 (15.7)	534 (17.6)	27 (18.0)	198 (21.2)	2427 (16.4)
Mean (95% CI) body mass index					
At age 25 years	22.1 (22.0, 22.2)	25.0 (24.8, 25.1)	34.0 (33.2, 34.8)	34.7 (34.4, 35.1)	23.5 (23.4, 23.6)
At 10 years before survey	24.6 (24.5, 24.6)	34.0 (33.8, 34.1)	27.0 (26.5, 27.5)	38.8 (38.3, 39.3)	27.1 (27.0, 27.3)
Mean (95% CI) absolute weight change, kg	6.2 (6.1, 6.4)	23.5 (22.9, 24.1)	− 19.1 (− 22.2, − 16.0)	10.5 (9.1, 11.9)	9.4 (9.1, 9.6)

All estimates accounted for complex survey designs

^a^At start of follow-up

^b^At end of follow-up

Regarding life-course weight change, 75.2% of the participants were in the stable non-obesity group, 5.9% were in the stable obesity group, 18.0% of the participants moved from non-obesity to obesity and they gained 23.5 kg on average, whereas only 0.9% of the participants moved from the obesity to non-obesity category and they lost 19.1 kg on average.

### Relations of weight change patterns with incident asthma

**Fig. 1:**
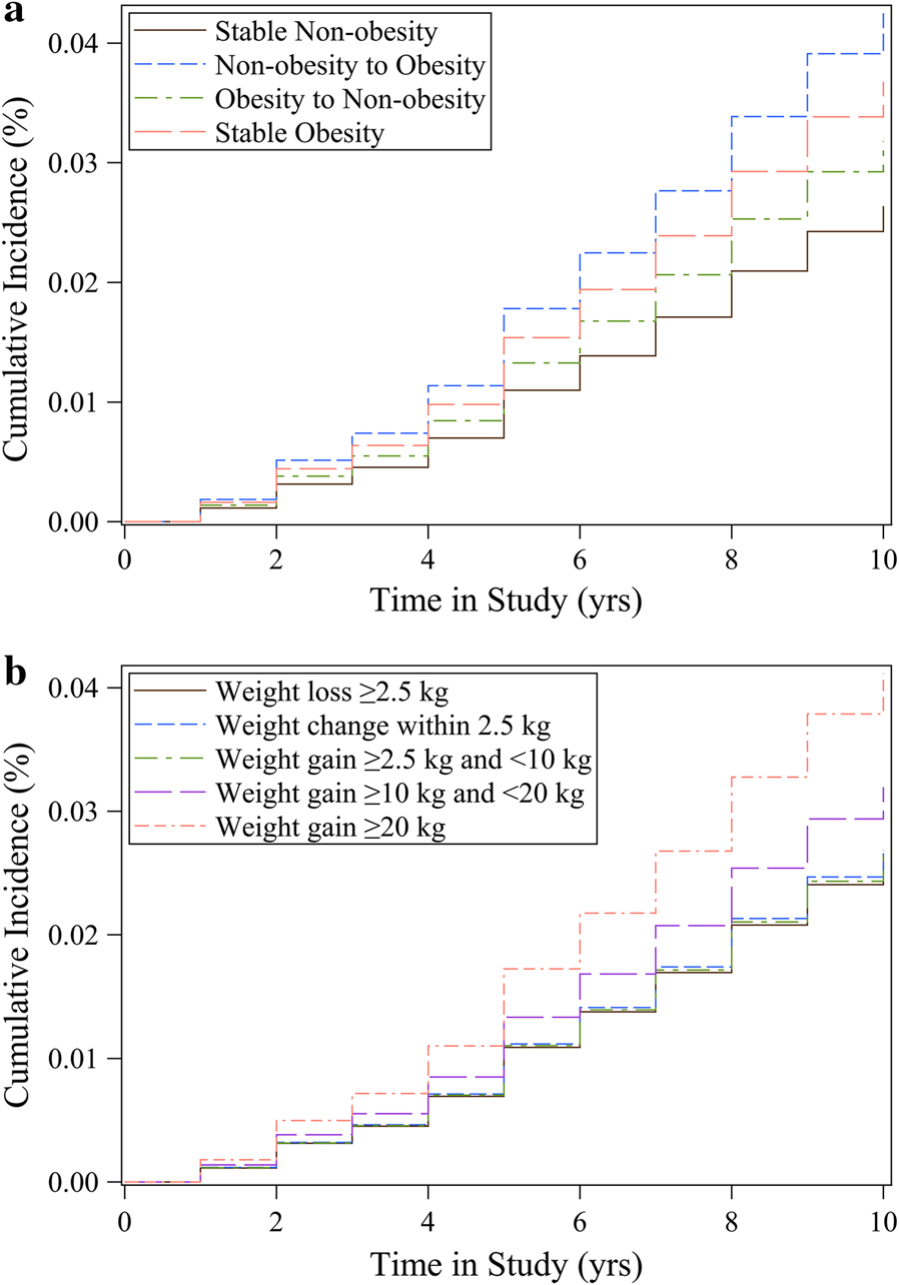
Cumulative incidence curve for asthma. Multivariable Cox regression model adjusted for baseline age, gender, race/ethnicity, education level, family income-poverty ratio level, smoking status, and family history of asthma. **a** Stable non-obesity, non-obesity to obesity, obesity to non-obesity, stable obesity. **b** Absolute weight change

Among 20,771 participants, 627 had a diagnosis of asthma, yielding an overall incidence rate of 3.1 per 1000 person-years. Figure 1 presented cumulative incidence curves by time in study for each weight change group. The cumulative incidence in the weight gain pattern (non-obesity to obesity / weight gain ≥ 20 kg) were highest. Compared with stable non-obesity individuals, stable obesity participants had increased risks of incident asthma during the 10 years of follow-up, with HR of 1.41 (95% CI = 0.97 to 2.05,* P* = 0.075) (Table 2). Moving from the non-obesity range at age 25 years to the obesity range at baseline was associated with a 63% higher risk of incident asthma (HR = 1.63, 95% CI = 1.29 to 2.07,* P* < 0.001). Compared with those who maintained stable non-obesity from young adulthood through midlife, the obesity to non-obesity group was not significantly associated with incident asthma risk (HR = 1.21, 95% CI = 0.41 to 3.62, *P* = 0.730). The population attributable fractions were calculated to estimate the percentage of asthmas that could be averted under hypothetical scenarios. If the participants in the non-obesity to obesity group had not gained weight, 10.2% of the adult-onset asthmas would have been averted in U.S. adults. Furthermore, 12.7% of the adult-onset asthmas would have been averted, if the total population had a stable non-obesity pattern from young adulthood through midlife.

**Table 2:** Hazard ratios (95% CIs) of incident asthma with weight change patterns across adulthood in NHANES 1999–2016

Weight change patterns	No. of incident asthma/person-years	Model 1^a^		Model 2^b^	
		HR (95% CI)	*P*	HR (95% CI)	*P*
Category 1^c^					
Stable non-obesity	402/151,360	1		1	
Non-obesity to obesity	165/39,485	1.66 (1.30, 2.11)	< 0.001	1.63 (1.29, 2.07)	< 0.001
Obesity to non-obesity	7/2067	1.47 (0.50, 4.27)	0.479	1.21 (0.41, 3.62)	0.730
Stable obesity	53/12,305	1.48 (1.02, 2.16)	0.041	1.41 (0.97, 2.05)	0.075
Category 2^d^					
Weight loss ≥ 2.5 kg	33/11,163	1.01 (0.60, 1.72)	0.964	0.91 (0.53, 1.56)	0.725
Weight change within 2.5 kg	126/48,635	1		1	
Weight gain ≥ 2.5 kg and < 10 kg	180/66,293	0.95 (0.70, 1.30)	0.744	0.99 (0.73, 1.34)	0.938
Weight gain ≥ 10 kg and < 20 kg	139/46,712	1.15 (0.85, 1.56)	0.372	1.19 (0.88, 1.60)	0.253
Weight gain ≥ 20 kg	149/32,414	1.52 (1.14, 2.03)	0.005	1.53 (1.15, 2.03)	0.004

All estimates accounted for complex survey designs

^a^Model 1 was adjusted for baseline age, gender, race/ethnicity

^b^Model 2 was additionally adjusted for education level, family income-poverty ratio level, smoking status, and family history of asthma. In the Category 2 analyses, we also included BMI at the age 25 years as potential confounders in model 2

^c^Stable non-obesity, BMI_age 25_ < 30 kg/m^2^ and BMI_10 years prior_ < 30; non-obesity to obesity, BMI_age 25_ < 30 kg/m^2^ and BMI_10 years prior_ ≥ 30; obesity to non-obesity, BMI_age 25_ ≥ 30 kg/m^2^ and BMI_10 years prior_ < 30; stable obesity, BMI_age 25_ ≥ 30 kg/m^2^ and BMI_10 years prior_ ≥ 30

^d^Absolute weight change: weight loss of at least 2.5 kg, weight change within 2.5 kg, weight gain of at least 2.5 kg but less than 10.0 kg, weight gain of at least 10.0 kg but less than 20.0 kg, and weight gain of at least 20.0 kg

**Fig. 2:**
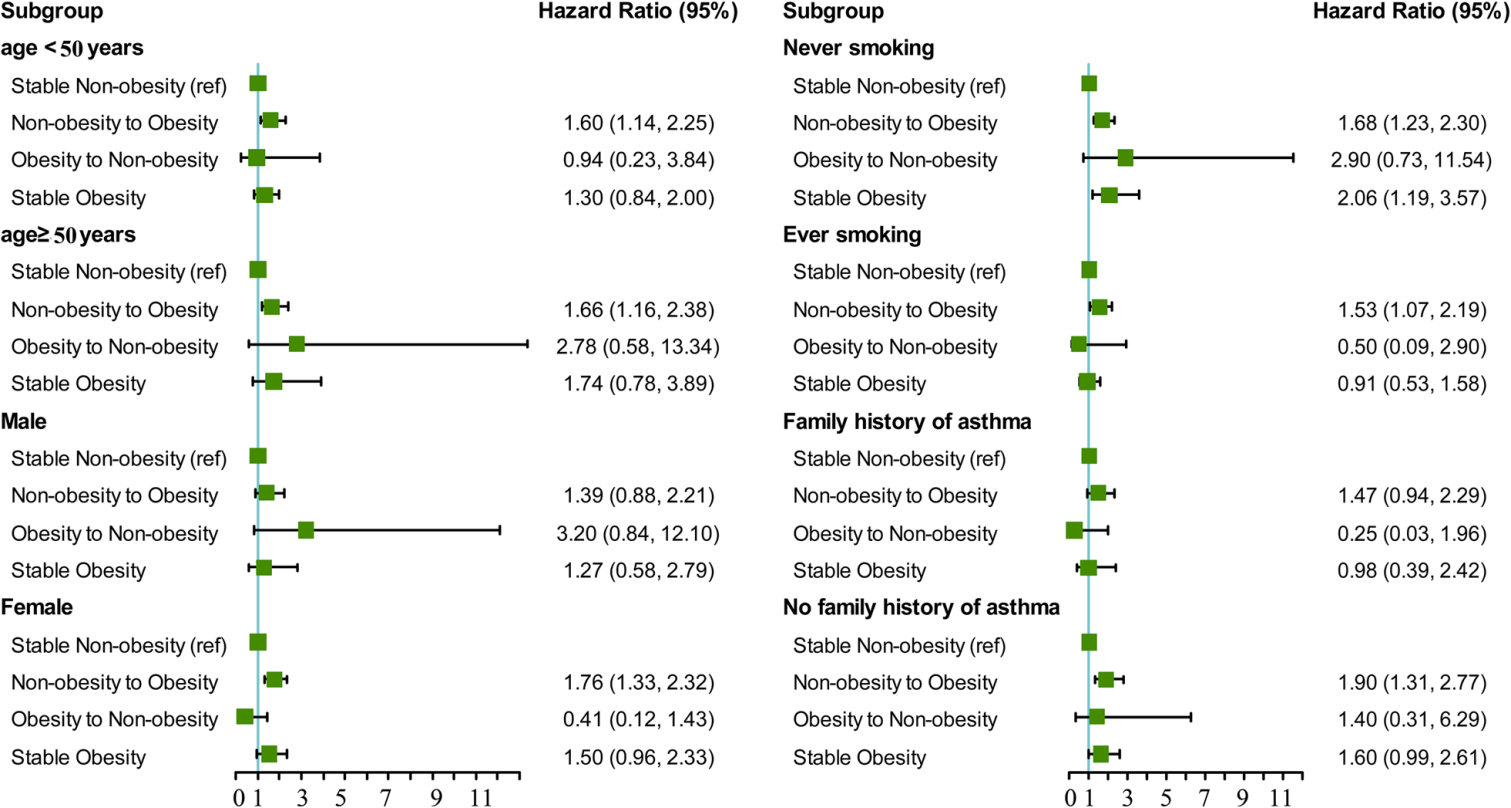
Associations between weight change patterns across adulthood and risk of incident asthma stratified by baseline age, gender, smoking status, and family history of asthma in NHANES 1999–2016. All estimates accounted for complex survey design of NHANES. Risk estimates were adjusted for baseline age (not adjusted in subgroup analysis by age), gender (not adjusted in subgroup analysis by gender), race/ethnicity, education level, family income-poverty ratio level, smoking status (not adjusted in subgroup analysis by smoking status), and family history of asthma. All *P* values for interaction were > 0.05

In the stratified analyses, the effects of weight gain (non-obesity to obesity) on the annual odds of developing asthma were more pronounced in female (HR = 1.76, 95% CI = 1.33 to 2.32, *P* < 0.001) than in male (HR = 1.39, 95% CI = 0.88 to 2.21, *P* = 0.159). The associations between weight gain and risk of incident asthma were stronger among participants who had no family history of asthma (HR = 1.90, 95% CI = 1.30 to 2.77, *P* < 0.001) compared with their counterparts (HR = 1.47, 95% CI = 0.94 to 2.29, *P* = 0.088) (Fig. 2). However, we found no significant interactions with baseline age, gender, smoking status and family history of asthma.

In the secondary analysis (Additional file 1: Table S1), compared with the stable normal group, the maximum overweight group was not significantly associated with incident asthma risk (HR = 1.19, 95% CI = 0.94 to 1.50,* P* = 0.139); stable obesity had a HR of 1.52 (95% CI = 1.03 to 2.25,* P* = 0.036) for incident asthma during the period; non-obesity to obesity pattern was consistently associated with highest incident asthma risk (HR = 1.77, 95% CI = 1.35 to 2.32,* P* < 0.001). Population attributable fraction calculation estimated that 21.8% of the adult-onset asthmas would have been averted, if the total population had a stable normal pattern from young adulthood through midlife.

When evaluating the absolute weight changes, the HRs for incident asthma in the extreme weight gain (weight gain ≥ 20 kg) group were 1.53 (95% CI = 1.15 to 2.03,* P* = 0.004) from age 25 years to baseline, compared with the stable weight group (weight change within 2.5 kg) (Table 2). Moderate to large weight gain (weight gain ≥ 10 kg and < 20 kg), small to moderate weight gain (weight gain ≥ 2.5 kg and < 10 kg) and weight loss (more than 2.5 kg) were not significantly associated with incident asthma. Furthermore, we identified a linear dose–response association between absolute weight change across adulthood and risk of incident asthma (*P* for trend = 0.024). These associations were independent of BMI at age 25 years. When we restricted the analysis to participants who had a BMI of < 25 at 25 years of age, results remained similar, suggesting that our estimates were not driven by participants with high BMIs who gained additional weight (data not shown). A similar does-response pattern was observed when absolute weight change was treated as continuous variables, for every 5 kg increase of the absolute weight change, the risk of incident asthma increased by 6% (HR = 1.06, 95% CI = 1.03 to 1.10, *P* < 0.001).

## Discussion

In this large retrospective cohort study of nationally representative U.S. adults, the highest risk of incident asthma was in participants who were weight gain (non-obesity to obesity) from early to middle adulthood; stable obesity across adulthood was also associated with increased risk of incident asthma. In addition, there was a dose–response effect of absolute weight gain on asthma incidence, independent of BMI in early adulthood. The effects of weight gain on the annual odds of developing asthma were more pronounced in female than in male. The findings underscored the importance of prevention of weight gain in early adulthood, for reducing incident asthma risk in later life.

Prior epidemiological data on the relationship between adulthood weight change and asthma were sparse. The California Teachers Study (CTS) including 88,304 women observed that weight gain ≥ 5 kg since age 18 years was statistically significantly associated with increased prevalence of adult-onset asthma [26]. However, weight gain did not clearly precede the observed asthma, these results may have simply reflected antecedent asthma followed by activity limitation and weight gain. By assessing weight change from the ages of 25 years to the baseline, the current study attempted to capture the changes in BMI during adulthood before the asthma starts, and to minimize reverse causation. In our study, weight gain from a non-obesity to an obesity pattern since young adulthood had a 63% higher incident asthma risk. Extreme weight gain (≥ 20 kg) from early to middle adulthood was associated with a 53% higher incident asthma risk. Higher weight gain predicted subsequent incident asthma in some [27–29], but not all [30], participants in the relevant prospective studies. Using data from the Nurses’ Health Study (NHS) with 85,911 female nurses aged 26–46 years at entry, Camargo et al. found that women who gained weight > 10 kg after age 18 were at significantly increased risk of developing asthma during the 4-year follow-up period [27]. Similarly, during a 3-year follow-up period of 67,229 women aged 40–65 years in the E3N Cohort Study, Romieu et al. reported that women who gained approximately ≥ 10 kg between the age of 20 years and baseline had a 89% increased risk of incident asthma [29]. Our results were consistent with these studies and indicated that avoiding obesity at young age and preventing weight gain from young to middle adulthood could be an important strategy to reduce future asthma risk.

We also found evidence that weight gain was related to incident asthma in women but not men. A number of population-based studies have suggested that the relationship between obesity and asthma may be stronger in females [31, 32]. Several possible mechanisms have been postulated to explain the obesity-asthma relationship. A sexual dimorphism exists in relation to body composition, in that females carry more fat subcutaneously and males carry more fat viscerally [31]. This led to distinct differences in the inflammatory pattern exhibited. For example, leptin is secreted more highly from subcutaneous adipose tissue and is therefore higher in females than males [31]. Compared to males, females have a smaller airway size relative to lung size [27]. An additional reduction in airway size caused by weight gain may disproportionately increase the susceptibility of females to asthma [7]. The increased estrogen levels associated with obesity are also thought to be one mechanism to explain the strong association between female obesity and adult-onset asthma [32, 33].

In our study, weight loss from young to middle adulthood was generally not significantly related to incident asthma compared with the stable normal group or stable obesity group. As weight loss from the obesity to the non-obesity among adulthood was rare, representing only 0.9% of the total population, and the results should be interpreted cautiously. More studies are needed to confirm the results in larger populations and explore the potential effect of weight loss among obesity people.

Adults gain weight more rapidly from young to middle adulthood, and excess adiposity mostly accrues in this period compared with the period from middle to late adulthood when weight begins to stabilize or even decrease [14]. In addition, preventing weight gain from young to middle adulthood might be more important than promoting weight loss, because achieving long term weight loss and maintaining it are difficult once a person becomes obese [14, 34]. Evaluating the long term effect of weight change, particularly weight gain from early to middle adulthood, on future health is thus important. Weight gain from early to middle adulthood is a well-established risk factor for diabetes, hypertension, cardiovascular disease, cancer, non-traumatic death, and many other diseases [14–17]. Our findings support adding asthma to this list and should provide yet one more piece of information to prevent weight gain and to support the aggressive implementation of public health measures to support the attainment of this goal.

We used population attributable fraction to explore the potential effect of prevention initiatives targeting weight gain. If all those who were non-obesity at age 25 prevent weight gain by midlife, 10.2% of observed incident cases of adult-onset asthma could be averted. In total, we found that 21.8% of asthma new cases during this time period could be reduced if the entire population maintained a weight in the normal range between early and middle adulthood in U.S. adults.

Our study had several strengths. Using a retrospective cohort design, we were able to take advantage of a large, nationally representative cohort of U.S. adults to estimate associations between weight change and incident asthma across the life course. As a nationally representative survey, results using NHANES are more broadly generalizable to the American population than those from other cohorts like the NHS and CTS. The advantage of focusing on weight gain throughout adult life is that it primarily reflects the accumulation of excess adiposity from early to middle adulthood, which is often ignored by individuals and their physicians because the consequences of modest weight accumulation may not yet be apparent. If the association between early adulthood weight gain and adult-onset asthma is proved to be causal, understanding and preventing early adulthood weight gain would be the next steps in research and translation, which is not only beneficial to future cardio-metabolic health, but also to mitigation of future asthma risk.

Our study also had several limitations. Firstly, we used recalled and self-reported weight data at age 25 years and 10 years before the NHANES survey and thus memory bias might have been introduced. However, a recent meta-analysis showed that recalled early life weight could be a valid measure to use in life course epidemiological analysis [35]. Secondly, we could not adjust for physical activity or diet because recall data on these variables were not collected. The results might thus partly reflect the effects of physical activity and dietary factors over the life course. Thirdly, members of the relevant cohorts who had died before the survey were not represented in the retrospective data set. Their experience might have differed from that of survivors in ways that affected the estimated relationship between obesity and asthma. Furthermore, our report relied on self-reported data on asthma status, which may have missed people who had not been diagnosed with the condition. Lastly, we did not evaluate the relations of changes in other adiposity related markers such as waist circumference and fat mass with incident asthma owing to lack of data at different time points. Further studies with repeated data on these markers may provide a more comprehensive picture of the changes in obesity status and asthma risk.

## Conclusions

Our study found that weight gain from young to middle adulthood was associated with increased risks of incident asthma in women but not men. Future studies are needed to unravel the mechanisms underlying the association between weight change across adulthood and adult-onset asthma, particularly the relations of changes in body composition to asthma. In addition, as weight loss was less achievable (only 0.9% participants changed from the obesity to the non-obesity category from early to middle adulthood), our results suggested that prevention of weight gain might be more important. If non-obesity at early adulthood prevented weight gain by midlife, approximately 10% of adult-onset asthma could be averted. Taken together, the findings indicated that maintaining normal weight throughout the adulthood, especially prevention of weight gain in early adulthood, should be encouraged to reduce risk of adult-onset asthma. Therefore, monitoring weight change since young adulthood could provide a sensitive and useful clinical measure for early detection of adverse trends in asthma risk.

## Supplementary Information

**Additional file 1: Table S1.** Hazard ratios (95% CIs) of incident asthma with weight change patterns for the secondary analysis. **Figure S1.** Survival analysis study design: weight change and onset asthmas. **Figure S2. **A flow chart of inclusion and exclusion of study participants.

## Data Availability

The datasets generated and analyzed during the current study are available at NHANES official website (https://wwwn.cdc.gov/nchs/nhanes/Default.aspx).

## Abbreviations

NHANES: National Health and Nutrition Examination Survey
NCHS: National Center for Health Statistics
CDC: Centers for Disease Control and Prevention
BMI: Body mass index
PAF: Population attributable fractions
CTS: California Teachers Study
NHS: Nurses’ Health Study
E3N: Etude Epidémiologique auprès de femmes de l’Education Nationale

## References

[1] Hales CM, Carroll MD, Fryar CD, Ogden CL (2020). Prevalence of obesity and severe obesity among adults: United States, 2017–2018. NCHS Data Brief..

[2] Akinbami LJ, Fryar CD (2016). Current asthma prevalence by weight status among adults: United States, 2001–2014. NCHS Data Brief..

[3] Beuther DA, Sutherland ER (2007). Overweight, obesity, and incident asthma: a meta-analysis of prospective epidemiologic studies. Am J Respir Crit Care Med.

[4] Skaaby T, Taylor AE, Thuesen BH, Jacobsen RK, Friedrich N, Møllehave LT, Hansen S, Larsen SC, Völker U, Nauck M (2018). Estimating the causal effect of body mass index on hay fever, asthma and lung function using Mendelian randomization. Allergy.

[5] Zhu Z, Guo Y, Shi H, Liu C-L, Panganiban RA, Chung W, O'Connor LJ, Himes BE, Gazal S, Hasegawa K (2020). Shared genetic and experimental links between obesity-related traits and asthma subtypes in UK Biobank. J Allergy Clin Immunol.

[6] Xu S, Gilliland FD, Conti DV (2019). Elucidation of causal direction between asthma and obesity: a bi-directional Mendelian randomization study. Int J Epidemiol.

[7] Brumpton B, Langhammer A, Romundstad P, Chen Y, Mai X-M (2013). General and abdominal obesity and incident asthma in adults: the HUNT study. Eur Respir J.

[8] Nystad W, Meyer HE, Nafstad P, Tverdal A, Engeland A (2004). Body mass index in relation to adult asthma among 135,000 Norwegian men and women. Am J Epidemiol.

[9] Burgess JA, Walters EH, Byrnes GB, Giles GG, Jenkins MA, Abramson MJ, Hopper JL, Dharmage SC (2007). Childhood adiposity predicts adult-onset current asthma in females: a 25-yr prospective study. Eur Respir J.

[10] Park S, Jung S-Y, Kwon J-W (2019). Sex differences in the association between asthma incidence and modifiable risk factors in Korean middle-aged and older adults: NHIS-HEALS 10-year cohort. BMC Pulm Med.

[11] Sonnenschein van der Voort AMM, Arends LR, de Jongste JC, Annesi-Maesano I, Arshad SH, Barros H, Basterrechea M, Bisgaard H, Chatzi L, Corpeleijn E (2014). Preterm birth, infant weight gain, and childhood asthma risk: a meta-analysis of 147,000 European children. J Allergy Clin Immunol.

[12] Chen YC, Chih AH, Chen JR, Liou TH, Pan WH, Lee YL (2017). Rapid adiposity growth increases risks of new-onset asthma and airway inflammation in children. Int J Obes.

[13] de Nijs SB, Venekamp LN, Bel EH (2013). Adult-onset asthma: is it really different?. Eur Respir Rev.

[14] Chen C, Ye Y, Zhang Y, Pan X-F, Pan A (2019). Weight change across adulthood in relation to all cause and cause specific mortality: prospective cohort study. BMJ (Clinical research ed).

[15] Stokes A, Collins JM, Grant BF, Scamuffa RF, Hsiao C-W, Johnston SS, Ammann EM, Manson JE, Preston SH (2018). Obesity progression between young adulthood and midlife and incident diabetes: a retrospective cohort study of US adults. Diabetes Care.

[16] Jia G, Shu X-O, Liu Y, Li H-L, Cai H, Gao J, Gao Y-T, Wen W, Xiang Y-B, Zheng W (2019). Association of adult weight gain with major health outcomes among middle-aged Chinese persons with low body weight in early adulthood. JAMA Netw Open.

[17] Zheng Y, Manson JE, Yuan C, Liang MH, Grodstein F, Stampfer MJ, Willett WC, Hu FB (2017). Associations of weight gain from early to middle adulthood with major health outcomes later in life. JAMA.

[18] Zipf G, Chiappa M, Porter KS, Ostchega Y, Lewis BG, Dostal J (2013). National health and nutrition examination survey: plan and operations, 1999–2010. Vital Health Stat.

[19] Berry KM, Neogi T, Baker JF, Collins JM, Waggoner JR, Hsiao C-W, Johnston SS, LaValley MP, Stokes A (2021). Obesity progression between young adulthood and midlife and incident arthritis: a retrospective cohort study of US adults. Arthritis Care Res (Hoboken).

[20] Zhou Y, Wang T, Yin X, Sun Y, Seow WJ (2020). Association of weight loss from early to middle adulthood and incident hypertension risk later in life. Nutrients..

[21] National Institutes of Health, National Heart, Lung and Blood Institute. Clinical guidelines on the identification, evaluation, and treatment of overweight and obesity in adults: the evidence report. NIH Publication No 98-4083. Washington, DC, 1998. https://www.ncbi.nlm.nih.gov/books/NBK2003/. Accessed 15 Feb 2021.

[22] Bundy JD, Mills KT, Chen J, Li C, Greenland P, He J (2018). Estimating the association of the 2017 and 2014 hypertension guidelines with cardiovascular events and deaths in US adults: an analysis of national data. JAMA Cardiol.

[23] SAS Institute Inc. SAS software version 9.4. Cary, NC, USA; 2014. http://www.sas.com. Accessed 15 Feb 2019.

[24] Gordon M, Lumley T. Forestplot: advanced forest plot using 'grid' graphics. R package version 1.10. 2020. https://CRAN.R-project.org/package=forestplot. Accessed 15 Dec 2020.

[25] R Core Team. R: a language and environment for statistical computing. R Foundation for Statistical Computing: Vienna, Austria; 2020. https://www.R-project.org/. Accessed 15 Dec 2020.

[26] Von Behren J, Lipsett M, Horn-Ross PL, Delfino RJ, Gilliland F, McConnell R, Bernstein L, Clarke CA, Reynolds P (2009). Obesity, waist size and prevalence of current asthma in the California Teachers Study cohort. Thorax.

[27] Camargo CA, Weiss ST, Zhang S, Willett WC, Speizer FE (1999). Prospective study of body mass index, weight change, and risk of adult-onset asthma in women. Arch Intern Med.

[28] Beckett WS, Jacobs DR, Yu X, Iribarren C, Williams OD (2001). Asthma is associated with weight gain in females but not males, independent of physical activity. Am J Respir Crit Care Med.

[29] Romieu I, Avenel V, Leynaert B, Kauffmann F, Clavel-Chapelon F (2003). Body mass index, change in body silhouette, and risk of asthma in the E3N cohort study. Am J Epidemiol.

[30] Hasler G, Gergen PJ, Ajdacic V, Gamma A, Eich D, Rössler W, Angst J (2006). Asthma and body weight change: a 20-year prospective community study of young adults. Int J Obes.

[31] Scott HA, Gibson PG, Garg ML, Wood LG (2011). Airway inflammation is augmented by obesity and fatty acids in asthma. Eur Respir J.

[32] Han Y-Y, Forno E, Celedón JC (2020). Sex steroid hormones and asthma in a nationwide study of US adults. Am J Respir Crit Care Med.

[33] Carey MA, Card JW, Voltz JW, Arbes SJ, Germolec DR, Korach KS, Zeldin DC (2007). It's all about sex: gender, lung development and lung disease. Trends Endocrinol Metab.

[34] Fontana L, Hu FB (2014). Optimal body weight for health and longevity: bridging basic, clinical, and population research. Aging Cell.

[35] De Rubeis V, Bayat S, Griffith LE, Smith BT, Anderson LN (2019). Validity of self-reported recall of anthropometric measures in early life: a systematic review and meta-analysis. Obes Rev.

