# Supplementary material for: Weight gain from early to middle adulthood increases the risk of incident asthma later in life in the United States: a retrospective cohort study
Source: Respir Res. 2021 May 5;22:139. doi: 10.1186/s12931-021-01735-7 (PMC8097961; doi:10.1186/s12931-021-01735-7)
Supplement: Supplementary file 1 — Additional file 1: Table S1. Hazard ratios (95% CIs) of incident asthma with weight change patterns for the secondary analysis. Figure S1. Survival analysis study design: weight change and onset asthmas. Figure S2. A flow chart of inclusion and exclusion of study participants. [file 12931_2021_1735_MOESM1_ESM.doc]

**Supplemental content**

**Weight gain from early to middle adulthood increases the risk of incident asthma later in life in the United States: a retrospective cohort study**

Tao Wang, PhD^a#^, Yunping Zhou, PhD^b#^, Nan Kong, MS^a^, Jianzhong Zhang, MS^a^, Guo Cheng, PhD^c^, Yuxin Zheng, PhD^a*^

a. School of Public Health, Qingdao University, Qingdao, Shandong, P.R. China.

b. School of Nursing, Qingdao University, Qingdao, Shandong, P.R. China.

c. Laboratory of Molecular Translational Medicine, Centre for Translational Medicine, Key Laboratory of Birth Defects and Related Diseases of Women and Children (Sichuan University), Ministry of Education, West China Second University Hospital, Sichuan University, Chengdu, Sichuan, P.R. China.

# Contribute equally

* **Corresponding Author:** Yuxin Zheng, School of Public Health, Qingdao University, 308 Ningxia Road, Qingdao 266071, China. Phone: 86-532-82991082. E-mail: [yxzheng@qdu.edu.cn](mailto:yxzheng@qdu.edu.cn).

**Table S1. Hazard ratios (95% CIs) of incident asthma with weight change patterns for the secondary analysis. ^a^**

| Weight Change Patterns ^b^ | No of incident asthma /person-years | Model 1 ^c^ | | Model 2 ^d^ | |
| --- | --- | --- | --- | --- | --- |
|  |  | HR (95% CI) | *P* | HR (95% CI) | *P* |
| Stable Normal | 202/74715 | 1 |  | 1 |  |
| Maximum Overweight | 200/76645 | 1.15 (0.91, 1.44) | 0.241 | 1.19 (0.94, 1.50) | 0.139 |
| Non-obesity to Obesity | 165/39485 | 1.77 (1.35, 2.33) | <0.001 | 1.77 (1.35, 2.32) | <0.001 |
| Obesity to Non-obesity | 7/2067 | 1.56 (0.53, 4.63) | 0.420 | 1.31 (0.43, 3.98) | 0.637 |
| Stable Obesity | 53/12305 | 1.57 (1.06, 2.33) | 0.024 | 1.52 (1.03, 2.25) | 0.036 |

^a^ All estimates accounted for complex survey designs.

^b^ Stable Normal pattern (<25.0 at both times), Maximum Overweight pattern (25.0-29.9 at either time but not ≥30.0 at the other time), Non-obesity to Obesity pattern (<30.0 at younger age and ≥30.0 later), Obesity to Non-obesity pattern (≥30.0 at younger age and <30.0 later), and Stable Obesity (≥30.0 at both times).

^c^ Model 1 was adjusted for baseline age, gender, race/ethnicity.

^d^ Model 2 was additionally adjusted for education level, family income-poverty ratio level, smoking status, and family history of asthma.

**Supplemental Figure**

**Figure S1. Survival analysis study design: weight change and onset asthmas.**

**Figure S2.A flow chart of inclusion and exclusion of study participants.**
